# The Big-Fish-Little-Pond Effect on the Four Facets of Academic Self-Concept

**DOI:** 10.3389/fpsyg.2018.01247

**Published:** 2018-07-20

**Authors:** Frances Hoferichter, Alexander Lätsch, Rebecca Lazarides, Diana Raufelder

**Affiliations:** ^1^School Pedagogy, Institute of Educational Science, University of Greifswald, Greifswald, Germany; ^2^Institute of Educational Science, University of Potsdam, Potsdam, Germany

**Keywords:** big-fish-little-pond-effect, social, criterial, absolute, individual academic self-concept (SESSKO), high-ability tracked students

## Abstract

The social context plays a decisive role in the formation of the academic self-concept (ASC) and has been widely studied as the big-fish-little-pond-effect (BFLPE). This effect describes that comparable talented students in high-achieving school settings have a lower ASC compared to equally talented students attending low-achieving settings. Past research has focused on students’ domain-specific ASC, while little is known about the relation of achievement-related classroom compositions and the various facets of ASC. Additionally, BFLPE-research has been critiqued to build its theoretical frame on social comparison theory, without providing sufficient empirical support. To address this gap, we analyzed how the single student’s social, criterial, absolute, and individual ASC relate to class-level achievement of 8th graders. Applying Multilevel Structural Equation Modeling (MLSEM) we found that all facets of ASC were significantly related to average-class achievement, while student’s social ASC revealed the strongest associated. The results reveal explicitly that average-class achievement is strongly related to social comparison processes.

## Introduction

The academic self-concept (ASC) has been established as one of the key constructs that determines academic success (Valentine et al., 2004) and shares vital structural characteristics with academic interest (Gogol et al., 2016). Following a rather broad definition, ASC is formed through academic-related experience and interpretations, combining attitudes, beliefs, and perceptions a student has about his or her own abilities and skills within the school context (Marsh and Hattie, 1996; Lent et al., 1997). Stressing the cognitive part of ASC, it is also defined as the entity of cognitive representations of one’s own abilities in academic achievement situations (Schöne et al., 2002).

Current research has widely shown that the domain-specific ASC of high-achieving students (e.g., in subjects such as math, languages, biology, and physics) is impacted by the composition of the class (Köller and Baumert, 2001; Dumont et al., 2017; Stäbler et al., 2017) as well as by the composition of the school (Harker and Tymms, 2004; Köller et al., 2006; for an overview see Marsh et al., 2008). However, little is known about the interrelations of achievement-related classroom compositions and the various facets of ASC, which are differentiated into social, criterial, absolute, and individual ASCs (Schöne et al., 2002). The present study addresses this gap by examining how these four facets of ASC are related to students’ overall grades at the class level.

## Academic Self-Concept and the Big-Fish-Little-Pond-Effect

As the ASC reflects a student’s beliefs about his or her scholastic abilities, it is closely and positively related to academic achievement, which was confirmed by numerous studies and described in previous reviews (West et al., 1980; Hansford and Hattie, 1982; Hattie, 1992; Zimmerman, 1995; Valentine et al., 2004). This solid finding is based on the rationale that individuals strive to maintain consistent self-views (Swann, 1997), according to self-affirmation and self-regulation theory (Steele, 1988; Bandura, 1989). Hence, students with high academic self-beliefs are more likely to show performance behavior or adjust their behavior accordingly (self-regulation) to increase their chances for academic success, while subsequent academic success confirms their high self-beliefs (self-affirmation). In this sense, the reciprocal effects model (Marsh, 1990a; Marsh and Martin, 2011) postulates that ASC and achievement mutually reinforce each other, which has been validated in numerous studies (Valentine et al., 2004; Arens et al., 2016).

Besides the impact of individual academic achievement on ASC, there is consent among researchers that social comparison impacts students’ ASC decisively (Markus and Wurf, 1987; Marsh and Craven, 2002; Zeinz and Köller, 2006). How social comparison mechanisms relate to ASC in academic settings is described by the big-fish-little-pond-effect (BFLPE), according to which comparable talented students who attend high-ability schools have a lower ASC compared to equally talented students who attend comprehensive schools (Marsh, 1990a; Seaton et al., 2009). Köller et al. (2006) explain this effect as follows: A student (little fish) who attends a high-ability class or school (big pond) has many opportunities for upward-comparisons, which in turn are related to a low self-concept. However, if a student (big fish) would attend a rather low-ability class or school (small pond), the opportunity for downward-comparisons increase, leading to an increase of the student’s self-concept. In fact, research has shown that the ASC of high-tracked students suffers a substantial decline, compared to students from lower-tracked schools (Becker et al., 2014; Arens and Watermann, 2015). This finding calls for further research of high-tracked students, as low self-concept impairs for example achievement, educational attainment, effort, and career aspirations (Valentine et al., 2004; Marsh and Martin, 2011; Nagengast and Marsh, 2012). Following this rationale, the current study focuses on students from high-tracked schools.

In the frame of social comparison, negative contrast as well as assimilation (or reflected glory) effects have been investigated as counterbalancing mechanisms in BFLP research. While school-average ability is related to a negative self-concept, as students compare themselves to brighter classmates with higher achievement (negative contrast effects), students of high-achieving schools feel pride in belonging to this type of school, which has a positive effect on their self-concept (assimilation effect or reflected glory effects). Research has shown that the BFLPE is the net effect of these counteracting mechanisms, while the negative contrast effect was shown to have a stronger impact, leading to an overall low self-concept of students (Marsh et al., 2000; Trautwein et al., 2009).

Since the beginning of BFLP research about 20 years ago, it has been speculated that the BFLPE would be a consequence of social comparison in high ability schools (Marsh, 1987; for review see Marsh et al., 2008). However, BFLP research has been critiqued for interpreting results in light of social comparison mechanisms, but lacking empirical proof for such underlying mechanisms (Dai and Rinn, 2008). As a response to this critique, Huguet et al. (2009) provide first empirical support of the role social comparison plays for the BFLPE: The study’s findings show that BFLPE is eliminated after controlling for students’ perceived relative standing in class and coexists with contrastive and assimilative effects (Huguet et al., 2009).

## Different Facets of Self-Concept and the Big-Fish-Little-Pond Effect

Current research on the BFLPE focuses on domain-specific mechanisms and often uses the Academic Self-Description Questionnaire (Marsh, 1990b) to measure ASC in 12–15 school subjects (Rindermann and Heller, 2005). Hence, most studies have disregarded measuring ASC in a format that represents general cognitive skills and academic achievement of students, independent of specific school subjects and grades. Most studies have also disregarded that students’ ASCs can be differentiated into different dimensions.

Schöne et al. (2002) developed a scale to assess ASC (SESSKO) that differentiates four non-domain-specific facets of ASC, namely social ASC (individual performance compared to performance of peers), criterial ASC (individual performance evaluated according to an objective criterion), absolute ASC (individual performance unrelated to any internal or external frames of reference), and individual ASC (individual performance compared to past performance). SESSKO items are related to aspects of giftedness, intelligence, learning ability, and the management of requirements (Schöne et al., 2002). It might be assumed that, at an individual level, all facets of ASC are positively related to previous achievement (Valentine et al., 2004). At the classroom level, it can be assumed that social ASC is mostly affected by average class achievement, as the BFLPE stresses the importance of social comparison. In this sense, social comparison theory postulates that there “exist[s], in the human organism, a drive to evaluate his opinions and abilities” (Festinger, 1954, p. 117). This pervasive social phenomenon is particularly powerful when individuals compare themselves to those similar to them (Suls et al., 2002; Huguet et al., 2009). Following this, it might be assumed that high-achieving students compare themselves to same-aged high-achieving students from their class by asking the question, consciously or unconsciously, “Am I as good as I ought to be?” (Suls et al., 2002, p. 159).

In contrast, the absolute ASC is not related to any frame of reference, which is explicitly mentioned by the authors (Schöne et al., 2002, p. 45). Hence, it can be assumed that the absolute ASC is not significantly related to the achievement of the class. Similarly to the general ASC, the absolute ASC captures general beliefs of students’ skills and abilities in school. In this sense, it is somewhat speculative whether the BFLPE holds for absolute ASC, as previous studies have yielded inconsistent results when examining general ASC. Becker and Neumann (2016) as well as Marsh and Craven (1997) did find evidence for the BFLPE, relating general ASC to class- and school-average ability respectively, but Jackman et al. (2011) did not find the BFLPE for university students attending a high-ability course. In fact, Marsh et al. (2008) argue that BFLPEs are stronger when using domain-specific effects, as they are more proximal compared to non-domain effects, which is the case for absolute ASC.

The assumptions about the individual and criterial ASC are similarly speculative: The criterial ASC represents students’ abilities and performance compared to school standards, so the frame of reference is rather abstract, lacking proximity to the social comparison process which is the essence of the BFLPE (Marsh et al., 2008). We therefore expect a small effect of average class achievement on criterial ASC.

Concerning individual ASC, students compare their actual performance within the high-ability class to their own previous performance. In our study, students were assessed 1.5 years after their transition from elementary to secondary school. Consequently, it might be assumed that students already knew their teachers and classmates and thus, were familiar with the average class achievement. Marsh et al. (2001) have shown that about 1 year after the school transition, students had adjusted to the new learning context and the BFLPE was present. Drawing on this finding, we expect to find the BFLPE by relating average class achievement to individual ASC.

## The Present Study

The present study investigates the BFLPE among students from high-ability-tracking secondary schools by relating class-level grades (averaged over Math, German, English as a Foreign Language, and Biology) to social, criterial, absolute, and individual ASC. In Germany students are tracked according to their achievement after 4th or 6th grade, depending on the federal state. Students with relatively high achievement are tracked into high-ability-tracking schools (Gymnasium), with the aim to obtain a diploma (Abitur) which will allow them to study at a University or College. All other students are tracked to secondary schools, in which they can obtain various kinds of certificates. Based on the reviewed empirical and theoretical rationale, we formulated and tested the following hypotheses:

(1) Overall achievement at the end of 7th grade are positively related to social, criterial, absolute, and individual ASC in 8th grade at the student level.
(2) The social ASC is strongly related to the average class achievement, supporting the assumption that social comparison mechanisms drive the BFLPE.
(3) The absolute ASC is weakly related to the average class achievement.
(4) It is speculative how criterial ASC is affected by average class achievement. However, based on the theoretical outline above, we expect a BFLPE to be present, relating average class achievement to criterial ASC.
(5) We expect the individual ASC to be impacted negatively by average class achievement.

## Materials and Methods

### Sample and Procedure

The analyses are based on a cross-sectional early adolescent sample (*N* = 779; *M_age_* = 13.09; *SD* = 0.50) in the federal state of Brandenburg, Germany. The participating students are from high-track schools (57% girls) in 47 classes, which were collected in 2011 at the beginning of 8th grade. In this study, entire classes were assessed, while the average number of students examined was 16.57 students per class.

After obtaining the formal approval from the government’s Department of Education, Youth and Sport of the federal state of Brandenburg to conduct the study, parents and/or legal representatives were asked for written informed consent so that students would be able to participate in the study. Afterward, the students themselves were asked for their consent to participate in the study. It should be noted that therefore self-selected biases could occur. However, the response rate was relatively high, namely 85%. The data was collected via anonymous, written, class-based questionnaires. At least two trained research assistants were present during each session to introduce the paper-pencil questionnaire format and to clarify any questions related to the items or the use of a Likert scale. To minimize potential difficulties with self-report data, operationalization and data-handling was executed following Chan’s (2009) suggestion that only well-established scales, for which construct validity was supported, should be used.

### Measures

#### Dependent Variables

In order to evaluate adolescents’ perceived ASC, we used SESSKO, developed by Schöne et al. (2002). SESSKO differentiates four facets of ASC measured on a five-point Likert scale: absolute ASC, individual ASC, criterial ASC, and social ASC. These scales were conceptualized for measurement of ASC in German speaking countries and validated on 3326 students (Grades 5–9): The absolute ASC showed the internal consistency of 0.83, and split-half reliability was 0.84, the individual ASC showed the internal consistency of 0.87, and split-half reliability was 0.88, the criterial ASC showed the internal consistency of 0.80, and split-half reliability was 0.81 and the social ASC showed the internal consistency of 0.88, and split-half reliability was 0.89.

(1) The *social academic self-concept* consists of six items and showed a satisfactory reliability in the present sample (αα = 0.92). This subscale focuses on the comparison of a student’s own abilities with that of other classmates, for example “I think I am more talented at school than my classmates” or “Learning new things is easier for me compared to my classmates.”
(2) The *absolute academic self-concept* consists of five items and showed a good reliability in the present sample (α = 0.88). This subscale is not related to any frame of reference model as it focuses on a rather general ASC including items such as “I am gifted at school” or “In school, many tasks are easy for me.”
(3) The *criterial academic self-concept* consists of five items and showed a good reliability in the present sample (α = 0.86). This subscale focuses on the ranking of an individual’s school achievement based on objective criteria, for example “When I look at what we have to do for school, I think I am talented” or “When I look at what we have to know in school, I think I am able to handle the tasks quite well.”
(4) The *individual academic self-concept* scale consists of six items and showed a good reliability in the present sample (α = 0.81). Questions focus on the comparison of a student’s own abilities in the past vs. the present and include items such as “Learning new things at school is easier for me than before” or “I am more intelligent than before.”

#### Independent Variables

To measure students’ achievement, students were asked to report their grades in four major subjects on their last report card (from the end of 7th grade): German, Math, English, and Biology. The German grading system ranges from 1 (very good) to 6 (insufficient). As grades were recoded, a high score indicates high achievement.

### Statistical Analyses

The following analyses were estimated using Mplus 7.2 (Muthén and Muthén, 1998–2012). Due to the nested structure of the data (i.e., students being clustered in classrooms), we used multilevel structural equation models (MLSEM) (Bouvaird, 2007). This method allows analyzing hierarchic, structured data-sets – in our case, students (individual-level) nested in classes (context-level) (Richter and Naumann, 2002). To analyze the impact of the educational context, we considered the classroom level and not the school level, as classrooms present the immediate context in which students learn and are therefore better suited to investigate the BFLPE (van Ewijk and Sleegers, 2010). To test the hypotheses, four MLSEMs were built to examine the associations between end-of-year grades and the four facets of ASC. Separate models were assessed to avoid possible suppression effects (Paulhus et al., 2004) as well as vertical multicollinearity, which may occur when analyzing correlated subscales that measure the same underlying attribute (Kock and Lynn, 2012). In each MLSEM, the independent variables (end-of-year grades) are manifest, whereas the dependent variables (four facets of ASC) are latent. Hence, the classroom-level construct (grades) is based on a formative aggregation of the student-level construct (see Marsh et al., 2009), which means that the aggregation is based on a group average of individual-grade characteristics. This is a common technique in various disciplines, such as educational science, sociology, and psychology (Marsh et al., 2009). Moreover, based on their simulation study with formative constructs, Lüdtke et al. (2008) have shown that when the sampling ratio was low, the models based on the latent-aggregation approach were suitable for formative variables as there was considerable sampling error. We chose to apply a latent-measurement/manifest-aggregation approach. The L2 ASC is latent as it is based on multiple indicators, but manifest regarding its aggregation from L1 to L2. In their simulation study, Lüdtke et al. (2011) have shown that this approach is very promising as it corrects error in multilevel data when the data provide only limited information in terms of the L2 construct, i.e., low ICC.

As recommended in most cases, if group differences are of interest, all parameters are group-mean centered, whereby only the in-group variance of the predictor variable is included in the prediction (Kreft et al., 1995; Richter and Naumann, 2002; Enders and Tofighi, 2007). Hence, group-mean centering allows interpreting the intercept as the expected outcome for a specific student in a specific classroom, whose covariate values are equal to the values of the specific classroom mean.

In order to investigate how classroom composition affects students’ achievement, separating the between-group and the within-group components from the total variation is a common procedure (Cronbach and Webb, 1975; Paccagnella, 2006). As the aim of this study is to examine the BFLPE, which examines frame-of-reference effects, separating the between-group and the within-group components is necessary, and therefore group-mean centering is appropriate. Missing data were completely at random, as Little’s MCAR test confirmed [χ^2^(12) = 15.81; *p* > 0.05], and therefore handled – by default – with full information maximum likelihood in Mplus.

## Results

**Table 1** shows the means, standard deviations (SD), minimum, and maximum of the variables of interest.

**Table 1:** Descriptive statistics (range, means, standard deviations).

Variables	Range	*M*	*SD*
Age	–	13.09	0.50
Grades	1–6	4.02	0.64
ASC_abs	1–5	3.49	0.03
ASC_ind	1–5	3.07	0.04
ASC_crit	1–5	3.42	0.03
ASC_soc	1–5	3.22	0.02


*Grades, averaged grades at the end of the school year 7 (Math, German, English as a foreign language, Biology) are recoded and range from 6 = very good to 1 = insufficient; ASC_abs, absolute academic self-concept; ASC_ind, individual academic self-concept; ASC_crit, criterial academic self-concept; ASC_soc, social academic self-concept.*

### Unconditional Models

Before we designed the final MLSEMs, we tested a serious of four separate unconditioned models for each facet of ASC, which included only the outcome variables. These models were built to provide information on the amount of variance at both levels (within and between), which are necessary to estimate the intraclass correlations (ICC). Whereas ICC1 estimates the total variance of individual students’ ratings, the ICC2 gives information about the reliability of classroom mean ratings (Lüdtke et al., 2009). **Table 2** shows the results of the ICCs. The ICCs are relatively low, however, it has to be mentioned that there are no standard values for acceptable reliability using ICC (Koo and Li, 2016). A low ICC-value can not only reflect the low degree of rater- or measurement-agreement, but also relate to the lack of variability among the sampled subjects, the small number of cases, or the small amount of raters being asked (Portney and Watkins, 1993; Lee et al., 2012). In case data provide only limited information (e.g., low ICCs and small number of groups) it is adequate to use the latent-measurement/manifest-aggregation approach, as it partially corrects for biased group effects (Lüdtke et al., 2011).

**Table 2:** Number of items, reliability (Cronbach’s α), ICC1 and ICC2 for dependent variables and example item.

*Scale*	*Numbers of Items*	α	*ICC1*	*ICC2*	*Example items on a 5 point Likert scale*
Grades	4	–	–	–	
ASC_abs	5	0.88	0.08	0.58	I am gifted for school.
ASC_ind	4	0.81	0.03	0.34	Learning new things at school is easier for me than before.
ASC_crit	5	0.86	0.02	0.25	When I look at what we have to do for school, I think I am talented.
ASC_soc	6	0.92	0.03	0.32	Learning new things is easier for me compared to my classmates.


*ICC1, Intraclass-correlations; ICC2, Reliability of aggregated construct at classroom level (climate-effect); Grades, averaged grades at the end of the school-year 7 (Math, German, English as a foreign language, Biology); ASC_abs, absolute academic self-concept; ASC_ind, individual academic self-concept; ASC_crit, criterial academic self-concept; ASC_soc, social academic self-concept.*

In sum, the unconditional models for social ASC [χ^2^_(23)_ = 193.06, *p* < 0.001; RMSEA = 0.09; CFI = 0.94; SRMR_within_ = 0.02, SRMR_between_ = 0.14], absolute ASC [χ^2^_(14)_ = 57.12, *p* < 0.001; RMSEA = 0.06; CFI = 0.97; SRMR_within_ = 0.03, SRMR_between_ = 0.08], criterial ASC [χ^2^_(14)_ = 76.74, *p* < 0.001; RMSEA = 0.07; CFI = 0.97; SRMR_within_ = 0.03, SRMR_between_ = 0.07], and individual ASC [χ^2^_(7)_ = 83.63, *p* < 0.001; RMSEA = 0.12; CFI = 0.95; SRMR_within_ = 0.04, SRMR_between_ = 0.08] showed satisfactory model fits.

After estimating the unconditional models, the four MLSEMs were computed. The model fit for the social ASC model [χ^2^_(33)_ = 149.80, *p* < 0.001; RMSEA = 0.07; CFI = 0.96; SRMR_within_ = 0.03, SRMR_between_ = 0.16], absolute ASC model [χ^2^_(22)_ = 69.51, *p* < 0.001; RMSEA = 0.05; CFI = 0.97; SRMR_within_ = 0.03, SRMR_between_ = 0.07], criterial ASC model [χ^2^_(22)_ 70.93, *p* < 0.001; RMSEA = 0.05; CFI = 0.98; SRMR_within_ = 0.02, SRMR_between_ = 0.06], and individual ASC model [χ^2^_(13)_ = 83.42, *p* < 0.001; RMSEA = 0.08; CFI = 0.95; SRMR_within_ = 0.04, SRMR_between_ = 0.10] each showed an acceptable fit to the empirical data.

### Multilevel Structural Equation Models (MLSEMs)

**Table 3** shows the associations between grades and the four facets of ASC.

**Table 3:** Results of the Multilevel Structural Equation Models with grades as independent variable and school self-concept components as dependent variables.

Variable	Model 1 (ASC_abs)		Model 2 (ASC_ind)		Model 3 (ASC_crit)		Model 4 (ASC_soc)	
	Group-Mean-Centered		Group-Mean-Centered		Group-Mean-Centered		Group-Mean-Centered	
				
	β	SE	β	SE	β	SE	β	SE
**Within (L1)**								
Grades	0.45***	0.05	0.30***	0.08	0.51***	0.05	0.54***	0.06
**Between (L2)**								
M_grades	0.18*	0.07	0.06	0.10	0.25*	0.09	0.07	0.07
**New parameters**								
betaC (context-effect)	-0.27*	0.09	-0.25*	0.12	-0.26*	0.10	-0.47***	0.09
zbetaC (centered)	-0.20**	0.06	-0.13*	0.06	-0.18*	0.07	-0.32***	0.06
*R*^2^ (L1)	0.20	0.04	0.04	0.02	0.21	0.03	0.23	0.04
*R*^2^ (L2)	0.25	0.17	0.04	0.15	0.45	0.22	0.19	0.40


*Level 1, student level (*N* = 779); Level 2, classroom level (*N* = 47); ASC_abs, absolute academic self-concept; ASC_ind, individual academic self-concept; ASC_crit, criterial academic self-concept; ASC_soc, social academic self-concept; Grades, averaged grades at the end of the school year 7 (Math, German, English as a foreign language, Biology); M_grades, aggregated mean-grades on L2; betaC, calculated context-effect (between-within); zbetaC, standardized context-effect by the SD of the predictor at L2 and the squared root sum of the criterion (Marsh et al., 2009); ^∗^*p* < 0.05, ^∗∗^*p* < 0.01, ^∗∗∗^*p* < 0.001.*

#### The Relationship Between Grades and Social Academic Self-Concept

**FIGURE 1:**
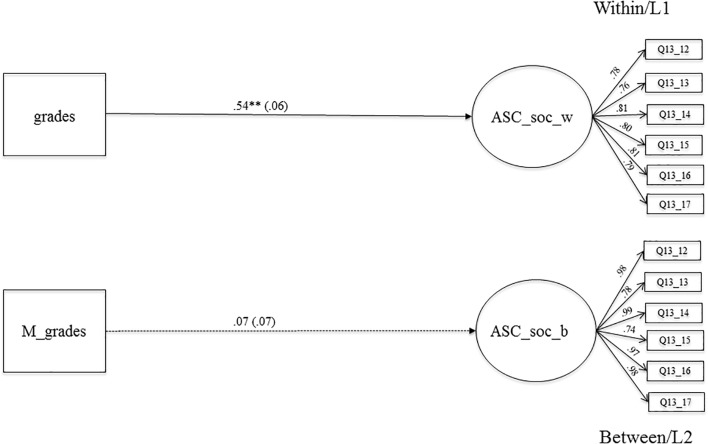
The relationship between averaged grades in Math, German, English as a foreign language. Biology and social academic self-concept on the within and between level. Level 1, student level; Level 2, classroom level; regression coefficients were standardized by group-mean-centering; grades, averaged grades at the end of the school year 7 (Math, German, English as a foreign language, Biology); ASC_soc_w, social academic self-concept on LI (within); M_grades, aggregated mean-grades on L2; ASC_soc_b, social academic self-concept on L2 (between); bold pathways are significant at *^∗^p* < 0.05, *^∗∗^p <* 0.001; dotted pathways are not significant.

**Figure 1** shows the results of the relationship between grades and social ASC. This association is positive and highly significant on the within-level (β = 0.54, *SE* = 0.06, *p* < 0.001). Students with higher grades at the end of 7th grade reported higher social ASC at the beginning of 8th grade. This association is not significant on the between-level, which means that classes with higher mean grades do not show higher levels of social ASC. However, the context effect (β = -0.47, *SE* = 0.09, *p* < 0.001), as well as the centered context effect (β = -0.32, *SE* = 0.06, *p* < 0.001), are negative and highly significant (see **Table 3**, Model 4), supporting the BFLPE (Marsh and Craven, 2002). The results of the centered context effect indicate that, with every increase of grades at the end of 7th grade by one SD, the social ASC decreases by almost one SD.

#### The Relationship Between Grades and Absolute Academic Self-Concept

**FIGURE 2:**
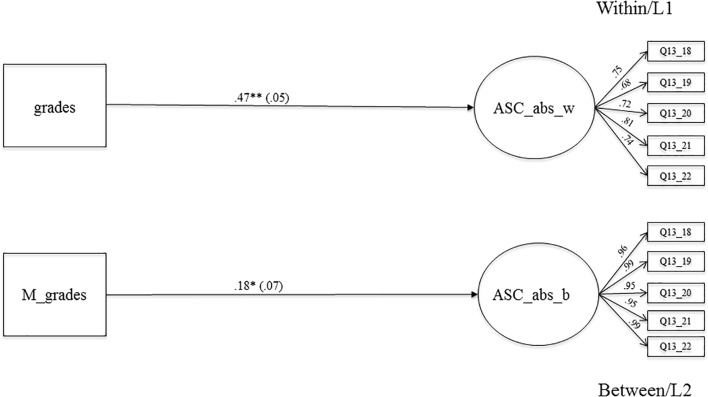
The relationship between averaged grades in Math, German, English as a foreign language. Biology and absolute academic self-concept on the within and between level. Level 1, student level; Level 2, classroom level; regression coefficients were standardized by group-mean-centering; grades, averaged grades at the end of the school year 7 (Math, German, English as a foreign language, Biology); ASC_abs_w, absolute academic self-concept on LI (within); M_grades, aggregated mean-grades on L2; ASC_abs_b, absolute academic self-concept on L2 (between); bold pathways are significant at *^∗^p* < 0.05, *^∗∗^p <* 0.001; dotted pathways are not significant.

**FIGURE 3:**
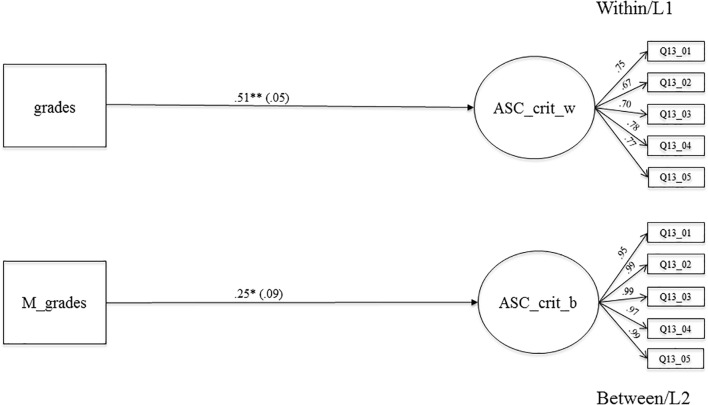
The relationship between averaged grades in Math, German, English as a foreign language. Biology and criterial academic self-concept on the within and between level. Level 1, student level; Level 2, classroom level; regression coefficients were standardized by group-mean-centering; grades, averaged grades at the end of the school year 7 (Math, German, English as a foreign language, Biology): ASC_crit_w, criterial academic self-concept on LI (within); M_grades, aggregated mean-grades on L2; ASC_crit_b, criterial academic self-concept on L2 (between); bold pathways are significant at *^∗^p* < 0.05, *^∗∗^p* < 0.001; dotted pathways are not significant.

**Figure 2** shows the MLSEM examining the relationship between grades and absolute ASC. The association is positive and highly significant on the within-level (β = 0.45, *SE* = 0.05, *p* < 0.001), which means that students with better grades at the end of 7th grade reported a higher absolute ASC at the beginning of 8th grade. In addition, results show a positive and significant association on the between-level (β = 0.18, *SE* = 0.07, *p* < 0.05), which means that classrooms with higher average achievement levels also show higher mean levels for absolute ASC. Moreover, the context effect (see in **Figure 3**, Model 1) is negative and significant (β = -0.27, *SE* = 0.09, *p* < 0.05). When comparing two students with the same grades, but from different classes, the students that were part of classes with higher grades showed a lower absolute ASC, supporting the BFLPE (Marsh and Craven, 2002). Moreover, the centered context effect was negative and significant (β = -0.20, *SE* = 0.06, *p* < 0.05), which means that with every increase of grades (by 1 *SD*) the absolute ASC of students decreases by 0.20 SDs.

#### The Relationship Between Grades and Criterial Academic Self-Concept

**Figure 3** shows the results of the relationship between grades at the end of 7th grade and criterial ASC at beginning of 8th grade. The association is positive and highly significant on the within-level (β = 0.51, *SE* = 0.05, *p* < 0.001), which means that students with higher grades also reported a higher criterial ASC. On the between level, the association is significant and positive (β = 0.25, *SE* = 0.09, *p* < 0.05), which means that classes with higher mean grades show higher mean levels for criterial ASC. Moreover, the context effect (β = -0.26, *SE* = 0.10, *p* < 0.05) and the centered context effect (β = -0.18, *SE* = 0.07, *p* < 0.05) (see **Table 3**, Model 3) are negative and significant. When comparing two students with the same grades, but from different classrooms, students from classes with a higher mean grade reported significantly lower levels of criterial ASC, supporting the BFLPE (Marsh and Craven, 2002).

#### The Relationship Between Grades and Individual Academic Self-Concept

**FIGURE 4:**
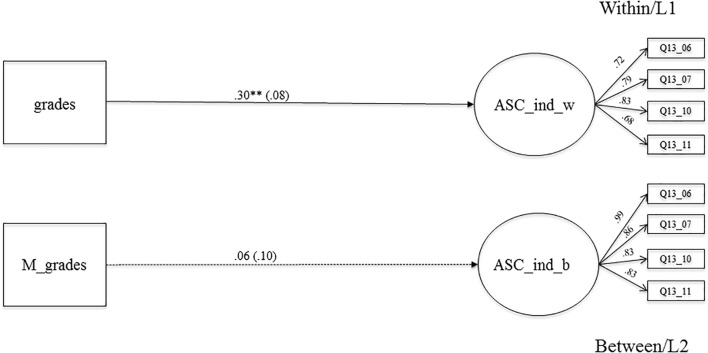
The relationship between averaged grades in Math, German, English as a foreign language. Biology and individual academic self-concept on the within and between level. Level 1, student level; Level 2, classroom level; regression coefficients were standardized by group-mean-centering; grades, averaged grades at the end of the school year 7 (Math, German, English as a foreign language, Biology); ASC_ind_w, individual academic self-concept on LI (within); M_grades, aggregated mean-grades on L2; ASC_ind_b, individual academic self-concept on L2 (between); bold pathways are significant at *^∗^p* < 0.05, *^∗∗^p <* 0.001; dotted pathways are not significant.

**Figure 4** shows the results of the MLSEM examining the relationship of grades and individual ASC. This association is positive and highly significant on the within-level (β = 0.30, *SE* = 0.08, *p* < 0.001), which means that students with better grades at the end of 7th grade reported a higher individual ASC at the beginning of 8th grade. This association is not significant on the between-level. This means that classes with higher mean grades do not significantly differ from classes with lower mean grades in their mean individual ASC. The context effect for this association (see **Table 3**, Model 2) is negative and significant (β = -0.25, *SE* = 0.12, *p* < 0.05). When comparing two students with the same grades, but from different classes, students from classes with a higher mean grade reported lower individual ASC, supporting the BFLPE (Marsh and Craven, 2002). This is also the case for the centered context effect (β = -0.13, *SE* = 0.06, *p* < 0.05) (see **Table 3**, Model 2).

Overall, the results show the BFLPE was present in our sample, as the context effects were significant for all facets of the ASC.

## Discussion

The current study investigates the BFLPE among high-ability-tracked students in 8th grade, relating average class-level grades to a student’s social, absolute, criterial, and individual ASC measured by SESSKO (Schöne et al., 2002). We thereby expand knowledge on the BFLPE, as so far no study has investigated the BFLPE on the various facets of cognitive ASC independent of school domains. The focus of the present study on different facets of students’ ASC is motivated by the fact that SESSKO measures cognitive representations of individual skills by using three facets that refer to frames of reference (social, criterial, and individual ASC) as well as absolute ASC without any frame of reference.

The results indicate that overall grades (the average grades in Math, German, English as a Foreign Language, and Biology) at the end of the previous school year were positively related to all facets of ASC at the student level, indicating that students with high achievement tend to have a high social, absolute, criterial, and individual ASC. Consequently, the first hypothesis was confirmed. These results are in line with previous studies, which found that academic achievement is positively related to self-concept (Becker and Neumann, 2016; Stäbler et al., 2017; for a review see Valentine et al., 2004).

Furthermore, the social ASC was shown to be strongly related to average class achievement, confirming the second hypothesis. When comparing two students with equal achievement, but from different classrooms, students from classrooms with higher overall achievement reported significantly lower levels of social ASC. This effect is particularly striking, because with every improvement of the overall grades on the class level, a student’s social ASC decreases. This result not only confirms the BFLPE in homogeneous high-achievement settings, but also supports the idea that the BFLPE is driven by social comparison mechanisms based on social comparison theory (Festinger, 1954; Suls et al., 2002). Hence, this study adds to BFLP research investigating social mechanisms (e.g., Huguet et al., 2009) and provides a response to the criticism of Dai and Rinn (2008, p. 286) that there is a “bulk of social comparison research that does not provide direct evidence regarding the BFLPE *per se*.”

The results further show that the absolute ASC was significantly affected by average class achievement, supporting the BFLPE and the third hypothesis (Marsh et al., 2001, 2007, 2008; Trautwein et al., 2006a,b). However, compared to social ASC, the absolute ASC was not as strong associated with the overall class achievement. This is in line with past research arguing that ASC that does not relate to a domain-specific frame of reference is not affected as strongly by the BFLPE (Marsh and Craven, 1997; Becker and Neumann, 2016).

Similarly, criterial ASC was significantly related to overall class achievement, though not as strongly as social ASC, supporting the fourth hypothesis, which was rather speculative. This may be explained by the lack of proximity of criterial ASC, asking students to compare their achievement to school standards. Hence, this small effect (compared to social ASC) may be explained by the lack of proximity to frames of reference, while the proximal nature of comparison processes is one postulate of the BFLPE. Concerning the individual ASC, the results indicate a significant relationship between class level achievement and individual ASC, supporting hypothesis five. Hence, as expected and in line with Marsh et al. (2001), the students in grade eight have adjusted to the new classroom setting that took place about 1.5 years ago.

In sum, if we assume that ASC is associated with social comparison mechanisms, which is the theoretical underpinning of the BFLPE (Marsh, 1990a; Marsh et al., 1995; Marsh and Rowe, 1996; Marsh and Craven, 2002; Zeinz and Köller, 2006), criterial as well as individual ASC may implicitly ask for social comparison mechanisms. At this point, the question arises whether a student’s evaluation of their own ability within the school context may ever occur without relating to the performance of his or her classmates, as learning and growing up takes place primarily in a social school context. In this sense, according to Rogers (1947), self-concept is a social product, formed and developed through social, interpersonal relationships.

### Strengths, Limitations, and Further Directions

This study is apparently unique as it is the first investigation of how class-average achievement is related to social, absolute, criterial, and individual ASC in the frame of BFLP research. The results reveal explicitly that class-average achievement are strongly related to social comparison processes. The findings complement research providing empirical support for social comparison mechanisms related to the BFLPE. Furthermore, in the current study, we examined whole classes. We were therefore able to tackle the BFLPE more precisely (van Ewijk and Sleegers, 2010) compared to studies that base their findings on only few students per class at the school level, using large, nationally representative samples.

Each study has its limitations, which in our case is the use of teacher-assigned grades and not standardized test results. As the current study was not part of a large, national representative study, no standardized tests were conducted. However, within our means we tried to handle grades in a way that they would represent students’ overall achievement by summing up end of year grades in different subjects assigned by different teachers, like Math, German, English as a Foreign Language, and Biology.

Furthermore, the cross-sectional nature of the data can be rated as a limitation as well as the sensitivity to self-selected biases due to the voluntariness to participate. However, as the current study aims to examine potential differences between the four facets of ASC, this design is adequate. Future longitudinal studies are warranted to confirm and expand these findings by identifying underlying mechanisms and processes. Additional research should also investigate how social-motivational mechanisms impact the BFLPE by investigating questions such as how the BFLPE is distinct among (a) students oriented toward mastery vs. performance goals (Butler, 1992), (b) students who belong to different socio-motivational types (Raufelder et al., 2013), or (c) students socialized in collectivistic and individualistic societies (McFarland and Buehler, 1995; Hoferichter et al., 2018).

## Author Contributions

FH wrote the main part of the paper. DR and AL did the statistical analyses. RL was mainly involved in the conceptualization, statistical advisement and helped reviewing the manuscript.

## Conflict of Interest Statement

The authors declare that the research was conducted in the absence of any commercial or financial relationships that could be construed as a potential conflict of interest.
